# Self-Supervised Contrastive Learning for Medical Time Series: A Systematic Review

**DOI:** 10.3390/s23094221

**Published:** 2023-04-23

**Authors:** Ziyu Liu, Azadeh Alavi, Minyi Li, Xiang Zhang

**Affiliations:** 1 School of Computing Technologies, RMIT, Melbourne, VIC 3000, Australia; ziyu.liu2@student.rmit.edu.au; 2 Coles, Melbourne, VIC 3123, Australia; liminyi0709@gmail.com; 3 Department of Computer Science, University of North Carolina, Charlotte, NC 28223, USA

**Keywords:** self-supervised learning, medical time series, deep learning, healthcare, pretext tasks, contrastive learning, systematic review

## Abstract

Medical time series are sequential data collected over time that measures health-related signals, such as electroencephalography (EEG), electrocardiography (ECG), and intensive care unit (ICU) readings. Analyzing medical time series and identifying the latent patterns and trends that lead to uncovering highly valuable insights for enhancing diagnosis, treatment, risk assessment, and disease progression. However, data mining in medical time series is heavily limited by the sample annotation which is time-consuming and labor-intensive, and expert-depending. To mitigate this challenge, the emerging self-supervised contrastive learning, which has shown great success since 2020, is a promising solution. Contrastive learning aims to learn representative embeddings by contrasting positive and negative samples without the requirement for explicit labels. Here, we conducted a systematic review of how contrastive learning alleviates the label scarcity in medical time series based on PRISMA standards. We searched the studies in five scientific databases (IEEE, ACM, Scopus, Google Scholar, and PubMed) and retrieved 1908 papers based on the inclusion criteria. After applying excluding criteria, and screening at title, abstract, and full text levels, we carefully reviewed 43 papers in this area. Specifically, this paper outlines the pipeline of contrastive learning, including pre-training, fine-tuning, and testing. We provide a comprehensive summary of the various augmentations applied to medical time series data, the architectures of pre-training encoders, the types of fine-tuning classifiers and clusters, and the popular contrastive loss functions. Moreover, we present an overview of the different data types used in medical time series, highlight the medical applications of interest, and provide a comprehensive table of 51 public datasets that have been utilized in this field. In addition, this paper will provide a discussion on the promising future scopes such as providing guidance for effective augmentation design, developing a unified framework for analyzing hierarchical time series, and investigating methods for processing multimodal data. Despite being in its early stages, self-supervised contrastive learning has shown great potential in overcoming the need for expert-created annotations in the research of medical time series.

## 1. Introduction

The widespread adoption of advanced wearable sensors and electronic records, both in-hospital and outside of it, has resulted in the generation of massive amounts of medical data [1,2,3]. Medical data encompasses a broad spectrum of data types that include unstructured data (e.g., demographics, administrative data, notes, medications, and billing records), laboratory tests (e.g., bodily fluids, pathology, microbiology examination), medical time series (e.g., heart rate and blood pressure), and images (e.g., MRI, fMRI, ultrasound images) [4,5,6,7,8]. In this systematic review, we investigate the medical time series data which are the sequential observations (e.g., physiological signals and vital signs) that are related to human health. These time series are typically measured quantitatively by a medical device and then analyzed by a physician or specialist to assess the patient’s current status [9]. Taking a step further, we mainly focus on the physiological or biomedical time series that can be measured in a short period of time (minutes to hours). Note, in this systematic review, we do not study the sparse health history such as Electronic Health Records (EHRs) because they are more sparse, irregular, and suffer from lack of structure. For example, previous studies show individuals only visit the hospitals five times each year [10], making the EHR contain very limited time points for sequential analysis (a patient only has 50 events in 10 years of EHR). In contrast, we pay more attention to dense medical time series such as vital signs where each recording contains hundreds of time points [11]. The deep sequential models will benefit more from the latter, while we also note that the models used in physiological time series can be easily extended to EHR.

With the rapid development of deep learning and computational resources, many have applied deep learning methods to enhance medical time series analysis and aid in medical decision-making. Some of these methods have gained great success in improving the performance of both physiological signals classification (e.g., cardiovascular disease detection [12], neurological disorder [13]) and forecasting (e.g., mortality [14], sepsis shock [15]).

However, the performance and implementation of existing deep learning methods applied in medical time series analysis are limited by the accessibility of well-annotated labels. Even though the research community benefits greatly from the vast amount of new data collected daily by professional medical devices or ubiquitous devices, the cumbersome process of labeling biomedical time series lags far behind their generation. Manual labeling of biomedical data and physiological signals requires experts with domain knowledge and years of training, who often only have the time and resources to annotate a small subset of the dataset. For example, the medical devices at ICU can automatically collect vital signs 24 h every day but the bedside team can only have time to annotate a very limited portion of the acquired data. Moreover, in some scenarios with multiple experts, it is common that the data are hard to annotate due to the disagreement across experts. Taken together, these issues lead to label scarcity and sparsity in medical time series datasets which is a major impediment to the employment of deep learning in this area.

To mitigate the data scarcity, self-supervised contrastive representation learning has been shown as a promising manner [16]. We note two mainstream self-supervised learning strategies: contrastive and generative [17]. In this review, our main focus is on contrastive learning-based recent development in medical time series analysis. Contrastive learning is an emerging self-supervised learning paradigm that contains the following steps: (1) augment time series samples to generate positive and negative pairs; (2) map the augmented samples to a latent space with non-linear encoder; and (3) optimize the encoder with loss functions calculated in the latent space (through maximizing the distance between the embeddings of negative sample pairs, while minimizing the embedding distance of positive pairs) [6,17,18,19,20,21]. The ‘self-supervised’ means it does not require the true labels of samples in the stage of model training. Self-supervised contrastive learning drawn much attention since the development of SimCLR [16] in 2020. The contrastive learning techniques, including SimCLR and its successors, have primarily been developed for image processing and rarely applied to time series analysis [22]. Given the different data modalities, some of the common image augmentations, such as color changes or rotation, may not be as relevant to time series data [23]. Consequently, extending contrastive learning paradigms to time series presents significant challenges, especially in the health domain with unique characteristics (e.g., low frequency and high sensitivity [24]). However, self-supervised contrastive learning has great potential to mitigate the challenge of label scarcity in medical time series.

This paper provides a comprehensive and systematic review of recent developments in self-supervised contrastive learning methods for healthcare applications, with a specific focus on medical time series, while previous literature reviews have touched on self-supervised models, they have not comprehensively covered healthcare applications, making this paper a valuable addition to the existing body of knowledge [17,25]. In addition, while some surveys have explored self-supervised methods in medical imaging [6,7], this paper uniquely focuses on medical time series, an area that has received limited attention despite its crucial role in health informatics. As the first review to bridge self-supervised contrastive learning and time series analysis in healthcare, this paper provides novel insights into this important and emerging area of research. Overall, this paper fills a significant gap in the literature and contributes to advancing the state-of-the-art in self-supervised learning for healthcare applications.

### 1.1. Self-Supervised Contrastive Learning

**Figure 1:**
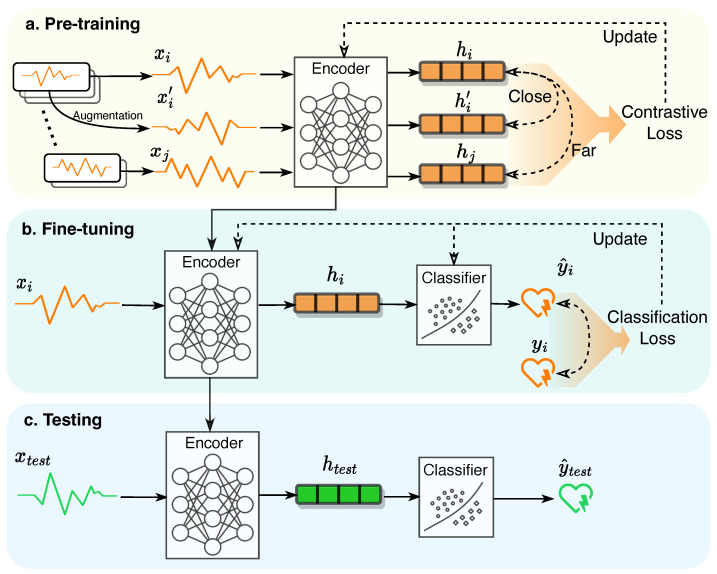
Pipeline of self-supervised contrastive learning which is composed of three stages. (**a**) The pre-training receives *unlabelled* time series sample $x_{i}$ as anchor sample, the augmented sample $x_{i}^{\prime }$ as positive sample (Section 3.4) while a different sample $x_{j}$ as negative sample. The $h_{i}$, $h_{i}^{\prime }$ and $h_{j}$ denotes learned embedding of the original sample $x_{i}$, positive pair $x_{i}^{\prime }$, and negative pair $x_{j}$, respectively. A contrastive loss (Section 3.7) is calculated based on the distance among embeddings of samples, which is used to update the encoder through backpropagation. (**b**) The well-trained encoder will be inherited by the fine-tuning stage which receives a *labeled* sample and makes a prediction through a downstream classifier. A standard supervised loss function (e.g., cross-entropy) will be used to update the encoder and/or classifier. (**c**) The testing stage makes predictions from the learned embedding $h_{\mathrm{test}}$ of an unseen test sample $x_{\mathrm{test}}$.

Next, we present the framework of self-supervised contrastive learning as shown in Figure 1. Contrastive learning contains three stages: pre-training, fine-tuning, and testing.

**Pre-training stage.** The pre-training refers to the process of self-supervised training the deep learning model (i.e., encoder) in contrastive manner while eliminating the dependency to the sample labels. In this stage, the encoder *f* receives a number of positive pairs (e.g., the original sample $x_{i}$ and the augmented sample $x_{i}^{\prime }$) and negative pairs (e.g., sample $x_{i}$ and a different sample $x_{j}$). Then, the encoder maps each sample (exampled by $x_{i}$) into a latent embedding space through

$$h_{i}=f\left(x_{i};\Theta \right)$$

where Θ denotes the model parameters. In the latent space, a contrastive loss L is used to measure the relative similarity across the embeddings:

$$L=\frac{\exp(\mathrm{sim}\left(h_{i},h_{i}^{\prime }\right))}{\frac{1}{N}\sum \exp\left(\mathrm{sim}\left(h_{i},h_{j}\right)\right)}$$

where sim() is a similarity function (e.g., cosine similarity), the smaller number detonates the two embeddings are more similar. The *N* denotes the batch size. By minimizing the loss function L, the model forces the positive samples to have close embeddings while the negative samples have far-away embeddings. More details and equations in Section 3.

**Fine-tuning stage.** The fine-tuning stage is composited of a well-trained encoder and a downstream classifier. The encoder’s parameters are inherited from the pre-training stage. This stage receives a time series sample $x_{i}$ and predicts an associated label $\hat{y}$. A classification loss can be calculated with predicted ${\hat{y}}_{i}$ and true label $y_{i}$. The loss is used to optimize the classifier (named partial fine-tuning when the encoder is frozen) or optimize both the encoder and classifier (named full fine-tuning). Please note the true label $y_{i}$ is required in fine-tuning stage, which means this stage is supervised learning. However, only small set of labeled samples are required to optimize the encoder and/or classifier because the encoder is pre-trained.

Let us have a concrete example to better understand the dataset in self-supervised contrastive learning. Suppose we have a dataset with 10,000 samples while only 5% have labels (i.e., 500 labeled samples). The traditional supervised learning cannot be trained with such a tiny labeled data size, however, with contrastive learning, we can use the 10,000 unlabeled samples to pre-train the encoder and then use the 500 labeled samples to fine-tune the model. Then, the model is likely to have great performance in the downstream task. The fine-tuning classification task is after the pre-training, that is why it is called ‘downstream’ task.

**Testing stage.** The testing stage is the same as the testing in machine learning: feed an unseen test time series $x_{\mathrm{test}}$ to the fine-tuned encoder and classifier to make the prediction.

Strictly speaking, contrastive learning comprises only the pre-training stage, which yields a well-trained encoder. However, to fully accomplish a task, fine-tuning and testing are indispensable. Thus, in this review, we mainly summarize the pre-training components while also providing a brief overview of fine-tuning and testing.

### 1.2. Systematic Review Objectives

The purpose of this systematic review is to comprehensively review the existing literature which adopts self-supervised contrastive learning to analyze time series data in a healthcare context. In order to facilitate researchers’ and readers’ understanding of this multidisciplinary field, we aim to provide clear and accessible navigation of potential solutions to the challenges in processing specific medical signals using contrastive learning methods. To this end, the research question and objectives addressed in this work are:

- What studies have been conducted in the intersection of self-supervised contrastive learning and medical time series analysis? See Section 3.1.
- Which specific types of medical time series have been investigated in the literature mentioned above? See Section 3.2.
- What healthcare scenarios or applications are commonly observed in this scope? See Section 3.3.
- How are contrastive learning models designed in terms of sample augmentation, pretext task, encoder architecture, and contrastive loss functions? See Section 3.4, Section 3.5, Section 3.6 and Section 3.7.
- Which public datasets are commonly used, and what are their statistics? See Section 3.8.
- What are the current challenges and future directions in this field? See Section 4.

## 2. Methods

We conduct a systematic review of self-supervised contrastive learning for analyzing medical time series following the Preferred Reporting Items for Systematic Reviews and Meta-Analyses (PRISMA) [26] guidelines.

### 2.1. Databases and Search Strategy

We searched for eligible literature in five databases including IEEE, ACM, Scopus, Google Scholar, and a medical domain specific database MEDLINE (PubMed). We gathered all the studies published before September 2022 with specific queries. The queries we used for each database are reported in Table 1. Initially, our search on MEDLINE retrieved only two relevant articles. To increase the comprehensiveness of our search and include as many relevant publications as possible, we modified the query on MEDLINE to remove some restrictions to cover more of the literature.

### 2.2. Eligibility Criteria

**Inclusion criteria.** The inclusion criteria are mainly based on the topic we have chosen and the research questions we want to investigate. Specifically, we select studies with the following properties: (1) involved the use of bio/medical signal; (2) adopt self-supervised contrastive learning for model training; (3) address a healthcare-related task; and (4) contain sufficient information to answer at least one of the questions listed in Section 1.2.

**Figure 2:**
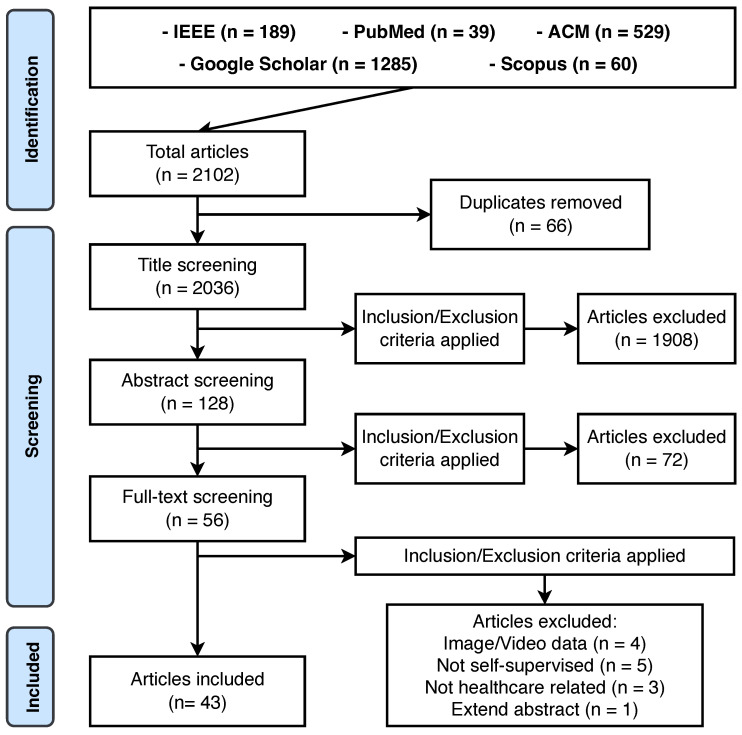
PRISMA diagram of the literature review process. We retrieved 43 publications among the 2102 papers that are collected from the five academic platforms.

**Exclusion criteria.** To begin with, we exclude duplicates, extended abstract, non-English, and irrelevant articles. There is a wide range of data types for studies in the interdisciplinary field of computer science and medicine, among them, we excluded studies with input as medical images-related data (e.g., MRI, fMRI, pathology image, retinal image, CT image) and Electronic Health Records (EHR) data. In addition, for studies in our scope and with the target data type, the ones were excluded if they engaged in a non-healthcare-related task (e.g., speech recognition). The PRISMA diagram summarizes the literature review process as shown in Figure 2.

## 3. Results

### 3.1. Overview

As shown in Figure 2, the database search returns 2102 papers in total. Based on the eligibility criteria (Section 2.2), we remove duplicated works, and conduct title screening, abstract screening, and full-text screening, respectively. The majority of papers (n = 1908) are removed as they are not developing nor applying self-supervised machine learning models. At last, there remain 43 papers for detailed review. We present the summary of the carefully reviewed studies in Table 2.

Based on the technical components in contrastive learning and the health-related tasks of the collected studies, we organize this review to elaborate on these research works from several perspectives, including the data type (Section 3.2), medical application (Section 3.3), augmentation (Section 3.4), pretext task (Section 3.5), pre-training encoder (Section 3.6.1), fine-tuning classifier (Section 3.6.2), contrastive loss (Section 3.7), public datasets (Section 3.8), and the model transferability and code availability (Section 3.9).

There are two mainstreams of deep learning-based self-supervised representation learning: contrastive (e.g., SimCLR [16]) and generative (e.g., VAE [27]) methods. In this systematic review, we mainly focus on contrastive learning which is more effective if the downstream task is classification [17]. However, we identify eight papers, which do not clearly fall into contrastive or generative categories, that is inspiring in the design of self-supervised framework. To enhance the diversity in the self-supervised learning community, we have summarized these papers in Table 3 and hope they will provide readers with valuable insights and inspiration.

### 3.2. Data Types of Medical Time Series

**Figure 3:**
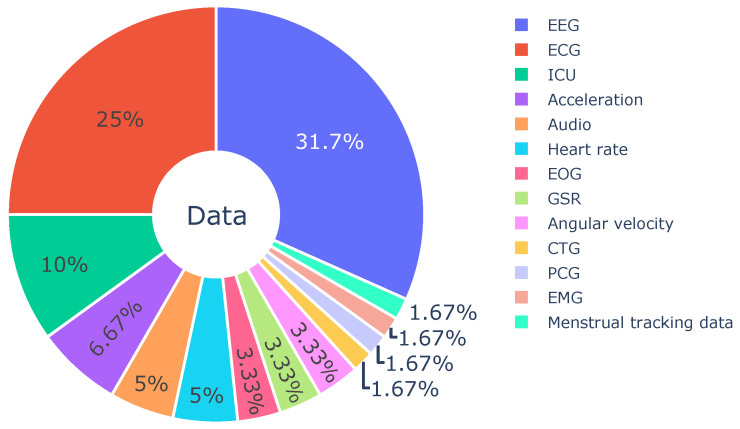
Types of medical time series in the reviewed papers. The majority of studies have focused on EEG, ECG, and ICU data, and one potential reason for this trend is the availability of large-scale public datasets in these fields. In contrast, other physiological signals may not have as many large-scale datasets available, making it more challenging to develop and validate machine learning models using those signals.

In this section, we summarize the physiological and biomedical time series types used in the reviewed publications and present the results in Figure 3. The majority of the reviewed studies used Electroencephalogram (EEG) or Electrocardiogram (ECG) as the input signal. One potential reason is that there are a number of publicly accessible large-scale datasets in EEG and ECG, indicating the fundamental infrastructures can greatly facilitate the research frontiers. In this section, we’ll first introduce the popular signals and then the understudied signal types.

**EEG.** In all the reviewed papers, we find 31.7% studies [20,21,28,29,30,31,32,33,34,35,36,37,38,39,40,41,42,43] used EEG. Among these, only one of these studies used intracranial EEG (invasive), and the others used non-invasive EEG. EEG detects the electrical impulse in the brain through numbers of electrodes set at certain spots on the scalp [44]. These electrodes link to a computer that will be able to record the sampled electrical impulse as one’s brain activities during a medical test or pre-defined task [45]. Although the aforementioned studies involved EEG as the same input signal, the tasks and scenarios are different in many aspects, which will be introduced in detail in application (Section 3.3).

**ECG.** In total, 25% of the reported studies [18,43,46,47,48,49,50,51,52,53,54,55] worked on ECG data. ECG (also known as EKG) is an effective and simple manner to assess the condition of the human heart. It also uses electrodes which place at specific locations on the chest to measure and record electrical activities of the heart. Of the studies we included in the ECG data category, most studies used a standard 12-lead ECG, while fewer leads (single, 2, or 4 leads) are also observed. It is worth mentioning that one study [56] used the abdominal ECG (aECG) as a non-invasive way to monitor the health of the fetus during pregnancy. The underlying assumption is that the aECG can be broken down into fetal and maternal ECGs. For another form of cardiac data that records sounds and murmurs, one work [57] adopted Phonocardiogram (PCG).

**ICU signals.** We found six studies that used ICU data (10%) [40,43,58,59,60,61]. ICU datasets typically include vital signs collected by professional medical devices in intensive care at a high time-sensitive level, as well as laboratory measurements and results, medications, admission diagnoses, procedures, chosen treatments, and more. The ICU signals are generally multivariate, unaligned, and sparse, thus it is challenging to achieve high performance in complex tasks (most contemporary studies work on relatively simple tasks).

**Audio signals.** A few works (5%) [38,62,63] adopted audio data, including the respiratory sound or breath, cough, and speech data.

**Heart rate and CTG.** Heart rate is the number of times the heart beats per minute. It is the most common physiological signal, which can be collected by professional medical equipment or smartwatches, and can also be derived from ECG data. Three works (5%) [39,64,65] adopted the heart rate data. Similarly, one study [66] used Cardiotocography (CTG) which is a temporal recording of both the fetal heart rate and uterine contractions. Heart rate or ECG may be included in ICU datasets.

**Acceleration and angular velocity.** Acceleration and angular velocity are often combined as the most common and effective data to describe human activities. They can be easily gathered by accelerometers and gyroscopes embedded in numerous devices such as smartphones and smartwatches. Two studies [39,67] adopted both signals as input, and another two works [64,65] only take the acceleration. Acceleration is one of the most popular and most affordable signals in human activity recognition (which may or may not relate to healthcare), we believe there will be more publications on acceleration analysis with contrastive learning.

**EOG**. Electrooculography (EOG) is similar to EEG but measures the electrical potential of eye movements instead of neuron activities. EOG data generally contain two channels that are collected using three electrodes (with a reference electrode). The electrodes are placed at the left and right sides of the eyes to measure the horizontal movements of the eyeballs. However, EOG is less popular than EEG and is mainly applied as auxiliary signal. In this systematic review, we only find two papers [38,39] that studied the self-supervised EOG analysis. Both of them use EOG along with EEG to formulate multi-modal datasets for the downstream tasks. It’s worth noting that [38] also collected Electromyography (EMG), which measures the electrical activity in muscle, to further enhance the dataset along with EEG and EOG: which is the only paper that explicitly mentioned self-supervised representation learning on EMG.

**GSR**. The galvanic skin response (GSR), also known as Electrodermal Activity (EDA), is a physiological signal that often accompanies ECG, heart rate, and EEG. GSR measures the changes in the electrical conductance of the skin, primarily through the sweat glands, as an additional indicator of emotional arousal levels or stimuli from the external world. In the context of the reviewed articles, two studies have incorporated GSR data into their experiments. Stuldreher et al. [43] analyzed the performance of clustering algorithms using EEG, heart rate, and GSR separately, as well as all possible combinations of the three modalities. Another study by Saeed et al. [39] performed self-supervised recognition of physiological stress using heart rate and GSR collected from real driving scenarios [68].

**Menstrual tracking data.** Last, but not least, we notice there is a study [69] that exploited the menstrual cycle tracking data from CLUE [70] to predict the discontinuation of birth control methods over time. Ref. [69] adopted self-supervised learning to address the challenges of both data imbalance and high sparsity.

All the public datasets of medical time series that were adopted in the reviewed studies were summarized in Table 4.

**Table 2:** Summary of self-supervised contrastive learning studies for time series analysis in healthcare. The studies are ordered by data type, applications, and data augmentations successively. The detailed explanations and summaries of each column are shown in Section 3.

Study	Data Type	Application	Augmentation	Pretext Task	Pre-Training Encoder	Fine-Tuning Classifier	Contrastive Loss	Datasets	Transfer?	Code?
Sarkar et al. (2021) [56]	Abdominal ECG	Maternal and fetal stress detection	Jittering; Scaling; Flipping; Temporal inversion; Permutation; Time-warping	Augmentation type recognition	CNN	MLP	Cross entropy	AMIGOS; DREAMER; WESAD; SWELL; FELICITy	✓	✓
Jiang et al. (2021) [67]	Acceleration, Angular velocity	Parkinson’s Disease detection	Rotation and permutations	Predictive coding	CNN + GRU	One-class SVDD	MSE	mPower study	-	-
Song et al. (2021) [63]	Audio	Respiratory sound classification	Jittering; Time shifting; Time stretching; Pitch shifting	Contrastive mapping	CNN	Logistic regression	NT-Xent	ICBHI 2017	-	-
Chen et al. (2022) [62]	Audio	COVID-19 detection	Block masking in time and frequency domains	Neighboring detection	CNN + Transformer	MLP	NT-Xent	DiCOVA - ICASSP 2022	-	-
de Vries et al. (2022) [66]	CTG	fetal health detection	-	Predictive coding	GRU + MLP	-	Cosine distance + triplet loss	Dutch STAN trial; A healthy dataset	-	-
Mehari et al. (2022) [46]	ECG	Cardiac abnormalities diagnosis	Time out; Random resized crop; Jittering	Predictive coding	LSTM	Linear layer	InfoNCE loss	CinC 2020; Chapman; Ribeiro; PTB-XL	✓	-
Li et al. (2022) [47]	ECG	Cardiac abnormality detection	-	Contrastive mapping	BiLSTM-CNN; TimeGAN	CNN	NT-Xent	MIT-BIH; PTB	-	-
Kiyasseh et al. (2021) [18]	ECG	Cardiac arrhythmia classification	Time-wise and channel-wise neighboring	Contrastive mapping	CNN	Linear layer	NT-Xent	PhysioNet 2020; Chapman; PhysioNet 2017; Cardiology	✓	✓
Luo et al. (2021) [48]	ECG	Cardiac arrhythmia classification	Reorganization	Detect organization operation	CNN	-	Cross-entropy	PhysioNet 2017; CPSC 2018	✓	-
Chen et al. (2021) [49]	ECG	Cardiac arrhythmia classification	Daubechies wavelet transform; Random crop	Contrastive mapping	ResNet + MLP	-	NT-Xent	PTB-XL; ICBEB 2018; PhysioNet 2017	✓	-
Wei et al. (2022) [50]	ECG	Cardiac arrhythmia classification	Trials discrimination	Contrastive mapping	Causal CNN	Logistic regression	Multi-similartiy loss	MIT-BIH; Chapman;	-	-
Kiyasseh et al. (2021) [51]	ECG	Cardiac arrhythmia clustering; Sex and age clustering	-	Detect clinical prototype	CNN	Linear layer	NCE loss	Chapman; PTB-XL	-	-
Nguyen et al. (2020) [52]	ECG	Cardiac arrhythmia detection	-	Predictive coding	LSTM-based autoencoder	MLP	MSE + Cross entropy	MNIST; MIT-BIH	-	-
Yang et al. (2022) [53]	ECG	Cardiac events diagnostic	Frequency masking; Croping and resizing; R-peak masking; Channel masking	Momentum contrast	ResNet	MLP	NT-Xent + KL-divergence	CPSC 2018;		✓
Mohsenvand et al. (2020) [28]	EEG	Emotion recognition; Seizure detection Sleep-stage scoring	Time shifting; Block masking; Amplitude scaling; Band-stop filtering; DC shifting; Jittering	Contrastive mapping	CNN	LSTM	NT-Xent	SEED dataset; TUH dataset; SleepEDF	-	-
He et al. (2022) [29]	EEG	Motor-Imagery classification	-	Predictive coding	CNN + LSTM	Linear layer	MSE	MI-2; BCIC IV 2a	-	-
Han et al. (2021) [71]	EEG	Motor-Imagery classification	Jittering; DC shift; Temporal roll, Amplitude scale, Temporal cutout, Crop and upsample	Contrastive mapping	CNN	CNN	NT-Xent	BCIC IV 2a	-	-
Wagh et al. (2021) [30]	ECG	EEG grade; Eye state; Demographics classification	Randomly flipping; Jittering	Hemipheric symmetry; Behavioral state estimation; Age contrastive	ResNet	-	Triplet loss	TUAB; MPI LEMON	-	✓
Xu et al. (2020) [20]	EEG	Seizure detection	Scaling transformations	Predicting transformation types	CNN	CNN	Cross-entropy	UPenn and Mayo Clinic’s seizure detection challenge	-	-
Ho et al. (2022) [31]	EEG	Seizure detection	Graph-based pair sampling	Contrastive mapping	GNN	Thresholding	NCE+MSE	TUSZ [72]	-	✓
Banville et al. (2019) [21]	EEG	Sleep scoring	Neighboring; Temporal shuffling	Contrastive mapping	CNN	Linear layer	Absolute distance	Sleep-EDF; MASS session3	-	-
Yang et al. (2021) [32]	EEG	Sleep stage classification	Bandpass filtering; Jittering; Channel flipping	Contrastive mapping	STFT + CNN	Logistic regression	Triplet loss	SHHS, Sleep-EDF, MGH Sleep	-	✓
Xiao et al. (2021) [33]	EEG	Sleep stage classification	-	Predictive coding	CNN + LSTM	Linear layer	InfoNEC loss + Cross-entropy	Sleep-EDF; ISRUC	-	✓
Ye et al. (2022) [34]	EEG	Sleep stage classification	-	Predictive coding	ResNet + GRU	Linear layer	InfoNEC loss	Sleep-EDF; ISRUC	-	✓
Jiang et al. (2021) [35]	EEG	Sleep stage classification	Crop + resize; Permutation	Contrastive mapping	ResNet	MLP	NT-Xent	Sleep-EDF; Sleep-EDFx; Dod-O; Dod-H	-	✓
Cheng et al. (2020) [36]	EEG, ECG	Motor-Imagery classification; Cardiac arrhythmia classification	Block masking with noise	Contrastive mapping	ResNet	Logistic regression	InfoNCE	Physionet Motor Imagery; MIT-BIH	-	-
Ren et al. (2022) [37]	EEG, ECG	Sleep stage classification; Cardiac arrhythmia classification	-	Predictive coding	MLP	CNN	Cross entropy	Sleep-EDF; MIT-BIH-SUP	-	-
Huijben et al. (2022) [38]	EEG, EOG, EMG, Audio	Sleep stage clustering; Speakers clustering	-	Predictive coding	CNNs	SOM	InfoNCE	MASS; LibriSpeech	-	-
Saeed et al. (2021) [39]	EEG, EOG, Heart rate, GSR, Acceleration, Angular velocity	Activity recognition; Sleep stage scoring; Stress detection; WIFI sensing	Permutating; Channel shuffling; Timewarp; Scaling; Jittering; etc.	Blend detection; Augmentation type recognition; Feature prediction from masked window; etc.	CNN	Linear classifier	Huber loss; MSE; Triplet loss; Cross-entropy	HHAR; MobiAct; UCI HAR; HAPT; Sleep-EDF; etc.	-	-
Zhang et al. (2022) [40]	EEG, ECG	Sleep disorder classification; Seizure detection	Jittering; Frequency masking; Time masking	Contrastive mapping	CNN	MLP/KNN	NT-Xent	SleepEDF; Eplipsy Seizure; HAR, etc.	✓	✓
Chen et al. (2021) [58]	ICU	Forecast adverse surgical events	-	Predictive coding	LSTM	LSTM	Cross-entropy	OR dataset; MIMIC dataset	✓	✓
Weatherhead et al. (2022) [60]	ICU	Mortality prediction; Diagnostic group classification; Circulatory failure prediction; Cardio pulmonary arrest prediction	Neighboring	Detect neighbors	Dilated causal CNN	LSTM	Min-max GAN loss	HiRID dataset; High-frequency ICU		-
Manduchi et al. (2021) [59]	ICU	Patient health state tracking	-	Predictive coding	VAE-LSTM	-	KL-divergence; Cross-entropy	MNIST; Fashion-MNIST; eICU dataset	-	✓
Ballas et al. (2022) [57]	PCG	Heart sound classification	High-pass filtering; Jittering + upsampling	Contrastive mapping	CNN	MLP	NT-Xent	PhysioNet 2016; PhysioNet 2022		-
Zhao et al. (2020) [65]	Acceleration, Heart rate, Bioradar, etc.	Sleep stage classification; Insomnia detection	Rotation	Rotation degrees recognition	CNN	Bi-LSTM- CRF	Cross-entropy	Sleep Bioradiolocation; PSG dataset; etc.	-	-

**Table 3:** Summary of self-supervised non-contrastive studies for medical time series. These studies do not strictly follow the framework of contrastive learning, but they can not be easily categorized because the paradigms are not standard. We list these studies here to increase the diversity of the self-supervised models and hope they can enlighten readers from broad fields. Apart from classification tasks, the Stuldreher et al. [43] adopt Kmeans for the downstream clustering.

Study	Data type	Applications	Model	Classifier	Loss	Datasets	Transfer?	Code?	Notes
Gedon et al. (2022) [54]	ECG	Cardiac abnormality detection	ResNet	Linear layer	MSE	Train: CODE; Test: CPSC 2018, PTB-XL	✓	-	Reconstruct the masked signal
Lee et al. (2021) [55]	ECG	Cardiac arrhythmia classification	ResNet	MLP	-	CPSC, PT-BXL, Chapman-Shaoxing	✓	-	-
Spathis et al. (2021) [64]	Hear rate, Acceleration	Personalized health-related outcomes prediction	CNN and GRU	Logistic regression	MSE; Quantile loss	The Fenland study	✓	✓	Sequence to sequence mapping
Tang et al. (2022) [41]	EEG	Seizure detection	DCGRUs	MLP	-	TUSZ, In-house dataset	-	✓	Forecast the future sequence
Yang et al. (2022) [42]	EEG	Seizure detection and forecasting	Conv-LSTM	MLP	Cross entropy	TUH seizure, EPILEPSIAE dataset, RPAH dataset (private)	-	✓	-
Stuldreher et al. (2022) [43]	EEG, EDA, ECG	Attentional engagement state clustering	PCoA+UMAP	Kmeans	-	Physiological Synchrony Selective Attention	-	-	Clustering
Wever et al. (2021) [69]	ICU, Menstrual tracking data	Mortality prediction, Discontinuation of birth control methods prediction	GRU-Decay	MLP	MaskedMSE	Physionet challenge 2012, Clue app data	-	✓	-
Edinburgh et al. (2020) [61]	ICU	Artefact detection on ICU physiological data	CNN-based VAE	-	MSE	Not mentioned (ABP waveform data from single anonymized patient throughout a stay)	-	✓	Reconstruct the signal

### 3.3. Medical Applications

**Figure 4:**
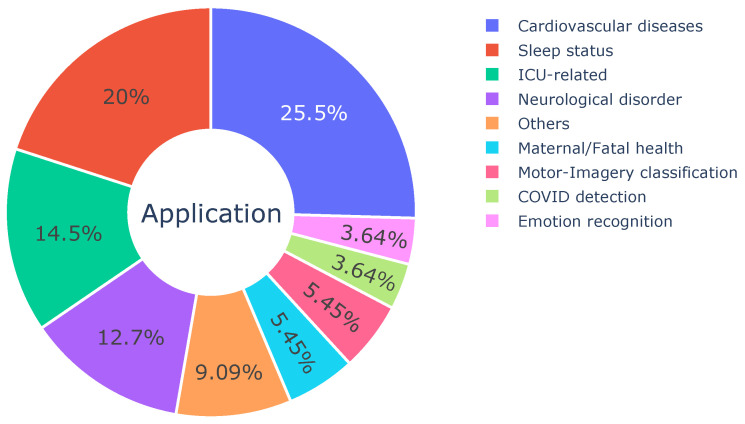
Applications of the reviewed papers. Consistent with the distribution of data types, the healthcare applications identified in this review predominantly focus on cardiovascular disease detection, sleep status monitoring, ICU-related scenarios, and neurological disorder diagnosis.

In this section, we summarize the health-related applications that have been applied as downstream tasks in the reviewed self-supervised contrastive learning algorithms. The distribution of medical applications is provided in Figure 4.

**Cardiovascular diseases.** Consistent with the distribution of data types, 25.5% of the reviewed studies performed experiments on cardiovascular disease-related detection/diagnosis. The specific applications mainly include cardiac abnormalities detection [46,47,53,54], cardiac arrhythmia detection or clustering [18,36,37,48,49,50,51,52,55], and heart sound classification [57]. Nearly all of the studies in this scope are based on ECG data, except one work [57] used PCG signals that record heart sounds and murmurs [73]. The reviewed studies on ECG abnormalities detection have focused on using self-supervised contrastive learning to distinguish between normal and abnormal ECG signals, and then applying the trained model to downstream tasks. While cardiac arrhythmia detection and classification share similarities with ECG abnormalities detection, the latter covers a broader range of heart events (e.g., conduction disturbance, myocardial infarction, hypertrophy, ST-T change, etc.), forms (e.g., abnormal QRS complex), and rhythms (e.g., arrhythmia) [46]. Most of the downstream tasks of these studies are based on binary or multi-class classification, and only one work [51] employed clustering and retrieves setting which creates clusters of similar patient attributes and enables the retrieve of associate information from it.

In comparison to cardiovascular applications, EEG-based scenarios have a broad range of applications across various domains such as sleep status monitoring, neurological disorder diagnosis, motor-imagery classification, and emotion recognition. EEG signals are highly sensitive to changes in brain activity and have thus emerged as a valuable tool in diverse fields including neuroscience research, clinical diagnosis, and the development of human–machine interfaces.

**Sleep status.** A large portion (20%) of research is related to sleep states [21,28,32,33,34,35,37,38,39,40,65], such as sleep stage scoring and sleep disorder classification (e.g., insomnia detection). Sleep stage can be categorized into five stages in accordance with the patterns of specific physiological signals (e.g., EEG, EMG, EOG): wake, non-rapid eye movement stage 1, non-rapid eye movement stage 2, non-rapid eye movement stage 3, and rapid eye movement stage [74]. The identification and annotation of these sleep stages often require manual intervention by trained professionals, as sleep assessment is an important indicator of an individual’s overall health. In the reviewed studies, self-supervised contrastive learning approaches were used to overcome the issue of label scarcity and enable the automatic classification of sleep stages. In the realm of sleep disorders, Zhao et al. [65] conducted insomnia detection based on bioradar data (continuous waves) from a non-contact sleep monitoring dataset [75]. The use of self-supervised learning in these studies enables the identification of sleep stages and disorders with greater accuracy and efficiency, which has the potential to improve overall patient care and health outcomes.

**Neurological disorder.** Neurological disorder detection/classification, accounts for 12.7% among all reviewed papers, which is another medical task that has recently gained significant attention within the field of self-supervised contrastive learning. However, the development of research in this branch is strictly constrained by the availability of data. For example, while Alzheimer’s dementia (AD), Parkinson’s disease (PD), Autism spectrum disorder (ASD), depression, and epilepsy seizure are all widely-spread neurological disorders, we found five studies on seizure detection [20,31,40,41,42], one on Parkinson’s disease detection [67] but zero on other diseases. The authors have consulted several experts in neuroscience and computer science, and note that the most potential reason for the imbalance across neurological diseases is the data availability. There are well-constructed infrastructures for epilepsy seizure (e.g., the TUH EEG Corpus [76]) but very limited public datasets on biomedical time series-based AD or ASD. It is worth mentioning that the PD dataset adopted by [67] is collected by smartphone when participants conduct different activities (e.g., memory, tapping, voice, and walking) [77], which is different from other reported papers that involved neurological disorder diagnosis. In [67], the main indicator is not EEG but human behavior data from accelerometers and gyroscopes that measures acceleration and angular velocity, respectively.

**Motor-Imagery classification.** Motion image classification is a growing field of brain–computer interface (BCI), which performs motor tasks only through imagination but without physical movements [71]. This is generally based on EEG as the main indicator and may have additional channels such as EOG or EMG to remove artifacts. By far, the motor tasks are still rather simple. For instance, the subject imagines to move the right finger or move the left hand, while the BCI system is collecting the subjects’ EEG signals and decode the signal to action intentions. However, this application can make big difference in rehabilitation engineering and understanding the neural mechanisms of cognitive neuroscience. Three (5.45%) of the reviewed studies [29,36,71] focused on EEG-based motor-imagery classification.

**Emotion recognition.** We include emotion recognition as the health-related task for potential applications in mental health and well-being. Two studies [28,39] employed emotion recognition as the downstream task, with one article [39] being closely related to the healthcare field by addressing stress detection using physiological data collected during real-world driving experiments. The use of self-supervised contrastive learning in emotion recognition tasks can lead to more accurate and efficient identification of emotional states, and aid in the development of interventions to improve overall health outcomes.

**ICU-related.** There is a large proportion (12.7%) among the reviewed papers focused on ICU-related tasks [58,59,60,61,69]. In this category, we include the tasks as long as it exploits one or multi-modal signals from ICU data [4], which comprise a number of tasks: mortality prediction, readmission after ICU discharge, length of stay in ICU, sepsis shock forecasting, etc. Chen et al. [58] used more than ten biomedical signals (blood oxygen saturation, end-tidal carbon dioxide, non-invasive blood pressure, fraction of inspired oxygen, end-tidal sevoflurane, ECG-derived heart rate, etc.) and six static variables (height, weight, age, gender, etc.) [78] for surgical adverse events forecasting. Similarly, Weatherhead et al. [60] applied the proposed unsupervised representation learning method on a high-time resolution ICU dataset [79] and used the learned embedding to train a simple network for three downstream medical tasks: 12-hour in-hospital mortality prediction, clinical diagnostic groups classification, and circulatory failure prediction. Moreover, the proposed architecture is also evaluated on a dataset from pediatric ICU for cardiopulmonary arrest prediction. Manduchi et al. [59] adopted an eICU dataset [80] which consists of multivariate medical time series, and calculated the Acute Physiology and Chronic Health Evaluation (APACHE) score. The APACHE [81] score is a widely accepted measure system of disease severity level that can be calculated from the physiologic vital signs, previous health status, and demographic information of the ICU patient. In light of the APACHE score, ref. [59] examined the proposed clustering method on four different labels (current severity score, worst future severity score in 6, 12, and 24 h) as the dynamic tracker of patient health. In contrast to the aforementioned studies focused on ICU-related applications, Wever et al. [69] addressed the class imbalance and missing value issues in time series analysis using the Physionet Challenge 2012 ICU dataset [82], a binary mortality classification dataset with the majority class representing over 85% samples and contains ∼80% missing data. Meanwhile, Edinburgh et al. [61] developed a self-supervised artifact detection algorithm for waveform physiological signals and evaluated it on arterial blood pressure (ABP) data from ICU. These studies demonstrate the potential of self-supervised contrastive learning to improve the performance of deep learning models on challenging clinical datasets with the class imbalance and missing data issues.

**Maternal/Fatal health.** Three studies [56,66,69] worked on a very interesting medical application: maternal and fetal health. Sarkar et al. [56] measured the abdominal ECG (aECG) which was further de-convoluted into fetal and maternal ECG. This study predicted the chronic stress of the mother based on hair cortisol, then estimates the fetal stress index and emotion of the fetus. De Vries et al. [66] took Fetal Heart Rate (FHR) and uterine contractions from Cardiotocography (CTG) to detect suspicious FHR events. Different from the status of the fetus, Wever et al. [69] developed a method to evaluate the discontinuation of birth control methods through the data collected from CLUE [70].

**COVID detection**. Affected by the outbreak of the pandemic, there are two publications aimed at detecting COVID-19 symptoms based on the sound of cough [62,83]. The novel techniques can promptly distinguish the acoustic signal of COVID-caused cough and the cough caused by other diseases (such as flu).

**Others**. Apart from the above applications, some works focused on a broad range of, but not concentrated, applications such as clustering the demographic (sex and age) of patients [30,51] and speaker clustering [38]. For simplification, we regard these studies as ‘others’ applications.

### 3.4. Augmentations

#### 3.4.1. Overview of Data Augmentation in Time Series

In self-supervised contrastive learning, data augmentation means to transform the *original sample*, through a designed manner, to an *augmented sample* that is derived from but slightly different with the original sample. The artificially generated samples can be used to provide a different aspect of the data. Importantly, the model can calculate the loss function by measuring the distance of embeddings between the original and augmented samples. The loss function is the so-called contrastive function that enables the back-propagation and empowers the whole model training. Thus, data augmentation is one of the most crucial components in contrastive learning.

Contrastive learning for time series data is still in the early stages of exploration, with ongoing developments and research. As a result, there is not yet a standard or unified approach to data augmentation in this field. Furthermore, some augmentation methods (e.g., rotation or adjusting pixels) are proposed in image processing but make less sense in time series. Researchers are actively experimenting with various types of augmentation methods to improve the performance of contrastive learning on time series data.

In this section, we comprehensively and systematically present the existing popular augmentation methods in time series. In particular, we will cover the augmentation method, including how the original sample is transformed into the augmented sample, positive pairs, which refer to pairs of samples with close embeddings, and negative pairs, which refer to pairs of samples with far-away embeddings. For better presentation, we define the following notations. For univariate time series, we denote the original sample as x which is a vector with *T* elements where each element is the observation $x_{t}$ (t≤T) at a specific timestep. We denote the augmented sample as $x^{\prime }$. For multivariate time series, the x and $x^{\prime }$ are matrices instead of vectors.

The x and $x^{\prime }$ are regarded as positive pairs as they are derived from the identical sample. Through a contrastive encoder *f*, the learned embeddings h=f(x) and $h^{\prime }=f\left(x^{\prime }\right)$ are as close as possible in the feature space. In opposite to the positive pair, a negative pair refers to two samples derived from different samples. For example, the $x_{i}$ and $x_{j}$, which are two samples from the dataset, form a negative pair as long as i≠j. The embeddings of the negative pair, such as $h_{i}$ and $h_{j}$, are as far as possible in the feature space. In this work, we summarize 16 commonly-used augmentations, and group them into three categories: transforming, masking, and neighboring.

#### 3.4.2. Transforming Augmentation

**Jittering**. Jittering, also known as adding random noise, is one of the most popular, simple yet effective augmentation methods [56]. In time series, jittering generates augmented sample $x^{\prime }$ by adding random noise to the original sample x. The random noise could follow a probability distribution as Gaussian, Poisson, or Exponential distribution, depending on the characteristics of the data and the noise. Gaussian noise is most commonly used.

**Scaling**. Scaling means rescale of the amplitude of the original sample [71]. For example, the range of the sample is [−1,1], after the transformation with a re-scale ratio of 1.5, the augmented sample will have the scale of [−1.5,1.5]. Note, the re-scale ratio could be different across time steps of the same sample and across different samples, so that the augmented dataset has higher diversity and is more robust to different variations.

**Flipping**. Flipping a time series means to reverse the order of time steps [56]. In other words, it is to reverse the order of elements in the time sequence. In math, for $x=\{x_{1},x_{2},\cdots ,x_{T-1},x_{T}\}$, the flipped sample will be $x^{\prime }=\left\{x_{T},x_{T-1},\cdots ,x_{2},x_{1}\right\}$.

**Permutation**. Permutation contains two steps: segmenting which splits the time series into several subsequences and permuting which randomly reorders the subsequences [35]. Each subsequence is a continuous subset of the original sample. Permutation is effective when the order of the data points is not important but the overall distribution of the data is.

**Time Warping.** It applies a non-linear transformation (a.k.a., warping) to change the timestamps (i.e., the time axis) of the time series [39]. In specific, it will stretch or compresses different parts of the time series. This is an important way to align the speed/duration of events addressing temporal distortions [84]. However, please note warping is not strictly an augmentation but a way to align multiple time series and calculate their distance/similarity more meaningfully.

**Time Shifting.** Time shifting means horizontally (along with the time axis) shifting the sample to generate the augmented sample [63]. For original sample $x=\{x_{1},x_{2},\cdots ,x_{T-1},x_{T}\}$, the shifted sample could be $x^{\prime }=\left\{x_{1+n},x_{2+n},\cdots ,x_{T-1+n},x_{T+n}\right\}$ where n is the shifting length. Empirically, we select the range of *n* in $\left[-\frac{T}{2},\frac{T}{2}\right]$.

**Resizing**. Resizing covers compressing and stretching, which alter the length of the time series while not changing the amplitude [46]. For original sample $x=\{x_{1},x_{2},\cdots ,x_{T-1},x_{T}\}$, we can compress the x with length T to a shorter time series (e.g., length $\frac{T}{2}$). A simple way to achieve the compression is downsampling, by taking an observation for every two values, so that the compressed sample $x^{\prime }=\left\{x_{1},x_{3},x_{5}\cdots ,x_{T-2},x_{T}\right\}$. Likewise, stretching means making the sample longer which can be achieved by interpolation that fills in missing observations using the mean value of neighboring observations.

**Slicing**. It randomly selects a subsequence of the time series as the augmented sample [49]. This augmentation is also known as cropping. For $x=\{x_{1},x_{2},\cdots ,x_{T-1},x_{T}\}$, the cropped sample is like $x^{\prime }=\left\{x_{1},x_{2},\cdots ,x_{T-m}\right\}$ where *m* is the number of time steps that are cropped out. As the sample length is reduced from *T* to T−m after slicing, generally the slicing augmentation is jointly used with resizing so that the augmented sample can have the same length as the original sample.

**Slicing + resizing.** It is similar to the augmentation of resizing [53]. It first selects a subsequence of the time series, then stretches it to the same size as the original sample.

**Rotation**. It is commonly used in computer vision but rarely in time series [39]. However, when you see rotation in time series augmentation, it means flipping the sample across the x-axis. In specific, it will times −1 on every observation. The rotated time series will be $x^{\prime }=\left\{-x_{1},-x_{2},\cdots ,-x_{T-1},-x_{T}\right\}$.

#### 3.4.3. Masking Augmentation

**Time masking.** It masks out some observations in the time series [71]. There are numerous modes for masking such as subsequence masking (masking a continuous period of the sample) and random masking (masking discrete data points). The masked observation values can be set as zero (zero-masking) or a different value (rescale-masking). This is one of the most common augmentation methods.

**Frequency masking.** Frequency masking is similar to time masking, but working on the frequency domain instead of time domain [40]. Generally, to perform frequency masking, we need to first transform the time series to frequency spectrum, through a transformation such as Fast Fourier Transform (FFT), and then mask out some components. Note, if applies zero-masking and subsequence masking in the frequency domain, the results will be the same as filtering (low-pass, band-pass, or high-pass).

**Filtering**. Filtering is a common method in signal processing, which means removing some unwanted components from the original sample [28]. Generally, filtering is conducted in the frequency domain to remove some frequency components. There are three ways of filtering: high-pass which removes the low frequency components, low-pass removes the high frequency bands, and band-pass filtering which removes all the frequency components except the specified bands. In biomedical time series, the high-pass (above 0.5 Hz) is most commonly used as the low frequency components are generally noises. Moreover, the power line frequency will be 50 Hz or 60 Hz based on different countries). The power frequency component needs to be notched out as it brings large noise from the data acquisition equipment/system instead of the physiological signals of interest. Please note that the filtering leads to the same results as band masking in the frequency domain.

**R-peak masking**. This is a subcategory of time masking but dedicated designed for ECG signal. It means to select the P-peak values (the highest observation and its neighbors) and mask them out [53]. As R-peak is informative in ECG signal, this augmentation forces the contrastive learning model to pay more attention to sub-informative patterns that might be overshadowed by the dominant R-peak.

#### 3.4.4. Neighboring Augmentation

**Time-wise neighboring**. Strictly, neighboring is not a kind of augmentation but a method to comprise positive pairs. It regards the two samples that are temporally near to each other as a positive sample [85]. The underlying assumption is that the temporal characteristic will not change dramatically, so the adjacent two samples should have similar embedding. For example, we have a time series $\hat{x}=\left\{x_{1},x_{2},\cdots ,x_{2T-1},x_{2T}\right\}$ with length of 2T. After segmenting the long time series into two samples with window length *T* and 0 overlapping, the output will be two samples: $x=\{x_{1},x_{2},\cdots ,x_{T-1},x_{T}\}$ and $x^{\prime }=\left\{x_{T+1},x_{T+2},\cdots ,x_{2T-1},x_{2T}\right\}$. Then x and $x^{\prime }$ are regarded as a positive pair; the negative pair will be the x and another sample that is far away from x.

**Channel-wise neighboring**. This is similar to Time-wise neighboring but considering the *spatial consistency* instead of *temporal consistency*. The underlying assumption is two channels that measure the same medical event will have similar embedding [18]. For example, two leads that monitor the same heartbeat will have similar embedding although they are placed at different positions of the chest.

### 3.5. Pretext Tasks

In contrastive learning, a pretext task is a task that is designed to help the model learn meaningful representations of the data in an unsupervised manner. The pretext task is not the final objective of the model but rather a way to provide the model with a meaningful and useful signal to learn from. The model is trained to solve the pretext task, and in the process, it learns to encode the data in a way that is useful for solving downstream tasks.

**Contrastive mapping.** Contrastive mapping, also known as contrastive instance discrimination, is the dominant pretext task in self-supervised contrastive learning models [16]. It’s not a strict ‘task’ as there is no specific task such as classification, but directly measures the relative distance of positive pair and negative pair in embedding space. By positive pair, we mean the pair of $\left(x,x^{\prime }\right)$ where x denotes the original sample (i.e., anchor sample) and $x^{\prime }$ denotes the augmented sample. The negative pair means the pair of x and other dissimilar pairs (such as the sample from a different patient).

The underlying assumption is that positive pairs (i.e., similar examples) should be close to each other in the embedding space, while negative pairs (i.e., dissimilar examples) should be far away from each other. Contrastive mapping transforms the samples from the original space to an embedding space in which the assumption is satisfied. We measure the contrastive loss in embedding space and aim to maximize the similarity between the features of positive pairs while minimizing the similarity between the features of negative pairs. By doing so, it encourages the feature representations to be distinctive and discriminative, which will benefit the downstream tasks. Note, the contrastive mapping must be used together with a contrastive loss (such as NT-Xent loss and NCE loss; Section 3.7) instead of a classification loss.

**Predictive coding.** This task is also called **autoregressive coding**. It trains an encoder to predict future observations based on past observations [86]. For example, we can design a predictive coding pretext task by mimicking the forecasting task: predict the value of $x_{T+1}$ for given $\left\{x_{1},x_{2},\cdots ,x_{T}\right\}$.

An important variant of predictive coding is to predict the correlation between the past and the future, instead of exactly predicting the future observation. In specific, the predictive coding asks the model to predict $d(x,x_{T+1})$ that denotes the distance between the embedding of x and $x_{T+1}$. The basic assumption is that $d\left(x,x_{T+1}\right)<d\left(x,x_{T+M}\right)$ where $x_{T+M}$ is temporally far away from x compared to $x_{T+1}$. In other words, the model is trained on positive pairs (consisting of the past data and the True next observation) and negative pairs (consisting of the past data and a different next observation). The positive pairs encourage the network to predict the correct next observation, while the negative pairs encourage the network to distinguish between different next data points.

**Neighbor detection.** This pretext task feed the pair of $\left(x,x^{\prime }\right)$ into the encoder. The x denotes the original sample while $x^{\prime }$ denotes a neighbor of x (see time-wise neighboring in Section 3.4 for details) [60]. However, different from the contrastive mapping, the pretext task of neighbor detection formulates the problem as a binary classification task: predict whether the input pair $\left(x,x^{\prime }\right)$ are neighbors or not. Accordingly, the loss will be measured by a classification loss such as cross-entropy.

**Trials discrimination**. Similar to neighbor detection, the pretext task of trial discrimination needs to recognize whether the two samples are from the same trial. A trial represents a *continuous* time series record, and generally, a sample is a subsequence of a trial. The basic assumption of the trial discrimination task is that two samples from the same trial will be more similar than samples from different trials due to inter-trial variations.

**Augmentation type recognition**. This is a flexible classification task aimed at determining whether a sample is the original or an augmented version [56]. It can be a binary classification task if only one augmentation is applied, or a multi-class task if multiple augmentations are applied simultaneously. For instance, a popular augmentation technique in computer vision is to identify the rotation angle of an image [25]. Similarly, bringing the idea to time series data, an intuitive pretext task is to predict whether the input sequence is permuted or not [87].

**Others**. Furthermore, there are a number of recently proposed pretext tasks that are interesting but not commonly used (most are only used in a single publication). We list them here for the reference of readers interested in details: momentum contrast [53], hemipheric symmetry [30], behavioral state estimation [30], age contrast [30], modality denoising [39], blend detection [39], feature prediction from masked window [39], fusion magnitude prediction [39], and clinical prototype detection [51].

### 3.6. Model Architecture

#### 3.6.1. Pre-Training Encoder

The ‘pre-training’ means the process of model training on the unlabeled dataset. It’s called ‘pre’-training because the training and testing (i.e., fine-tuning, Section 3.6.2) are two separate stages instead of an end-to-end framework. We first train the model until converges, then save the model parameters which will be loaded later for downstream task.

As shown in Table 2 and Table 3, the pre-training encoders are mainly composed of CNN and RNN (including GRU and LSTM). Note, each basic deep learning architecture (such as CNN, LSTM) has dozens of variations, we still regard the variations as the foundational model for simplicity. However, we discuss ResNet separately from CNN as ResNet is a milestone of the development of CNN and has its own fixed paradigm.

It is natural that lots of studies adopted LSTM as their backbone to build the encoder as LSTM is designed to process sequential data such as medical time series. However, it is not surprising to observe that CNN is also very popular because researchers empirically found that CNN (such as 1DCNN) can learn representative embeddings for time series. Apart from CNN and RNN, generative models such as VAE are also used in some papers for sample reconstruction.

#### 3.6.2. Fine-Tuning Classifier

Fine-tuning is a stage after pre-training, aiming at adjusting the model parameters to suit the specific dataset. In the context of contrastive learning, fine-tuning generally uses a proportion of labeled samples. The fine-tuning classifiers in the reviewed publications contains a variety of architectures including logistic regression [36], linear layer [46,56], CNN [21,71], LSTM [28], and MLP [35], etc. When the fine-tuning aims at clustering, the Kmeans [43] and SOM [38] are used to undertake the task.

There are mainly two ways to optimize fine-tuning classifiers: linear (freeze the parameters in the encoder) and fine-tuning (not freeze the parameters in the encoder). Note, here the ‘linear’ only means the pre-trained model parameters will not be updated, but the downstream classifier can be non-linear. Due to the confusion, we suggest calling the two streams of classifiers as *partial* and *full* fine-tuning (terminologies borrowed from the field of transfer learning).

### 3.7. Contrastive Loss

In this section, we mainly report the contrastive losses which can be calculated without the information of the true label. We do not elaborate on some loss functions in detail here because they are standard classification losses although they are mentioned in Table 2 and Table 3, such as cross-entropy and mean squared error (MSE), etc.

**NT-Xent loss**. The NT-Xent is the abbreviation of “Normalized Temperature-Scaled Cross Entropy Loss”. It’s an improved version of cross-entropy loss which is a widely used classification loss. The NT-Xent scales the logits with a small temperature coefficient, which helps to balance the confidence of the model in its predictions. The NT-Xent is very popular in contrastive learning as the SimCLR [16] adopted NT-Xent, which measures the difference between the similarity scores of a positive pair and all the negative pairs. The equation for NT-Xent can be written as

(1) $$L_{\mathrm{NT}\text{-}\mathrm{Xent}}=-\frac{1}{N}\sum_{i=1}^{N}\log\frac{\exp(s\left(x_{i},x_{i}^{\prime }\right)/\tau )}{\sum_{j=1,j\neq i}^{2N-1}\exp\left(s\left(x_{i},x_{j}^{\prime }\right)/\tau \right)}$$

where *N* is the number of samples in a mini-batch. The s(·) denotes the cosine similarity between two vectors and τ denotes the temporal scale factor (typically set as 0.5). In the denominator, the $\sum_{j=1,j\neq i}^{2N-1}\exp\left(s\left(x_{i},x_{j}^{\prime }\right)/\tau \right)$ denotes the summed cosine similarity of all the negative pairs. Here there are 2N−1 items because we have 2N samples for each batch including *N* original samples and *N* augmented samples.

By minimizing the NT-Xent loss, we encourage the model to learn a small $s(x_{i},x_{i}^{\prime })$ for positive pair but a large $s(x_{i},x_{j}^{\prime })$ for negative pair. Thus, after model convergence, the embeddings from positive pairs will be close to each other, while the embeddings from negative pairs will be far apart.

**NCE loss**. NCE is short for Noise Contrastive Estimation which approximates the true likelihood of the data by contrasting it with a negative sample [88]. In math,

(2) $$L_{\mathrm{NCE}}=-\frac{1}{N}\sum_{i=1}^{N}\log\frac{\exp(s\left(x_{i},x_{i}^{\prime }\right))}{\exp(s\left(x_{i},x_{j}^{\prime }\right))}$$

where $x_{i}^{\prime }$ is a similar sample with x while $x_{j}$ is a negative sample. NCE loss can be used with large amounts of data because the negative examples can be generated on the fly and do not need to be stored in memory. Compared to NT-Xent loss and InfoNCE loss, NCE has a simpler Equation (i.e., no accumulation in the denominator) and is computationally efficient, making it suitable for large-scale machine learning tasks.

**InfoNCE loss**. InfoNCE [86] is an extended version of NCE loss. InfoNCE is able to distinguish the positive sample from all the negative samples. The equation is as below:

(3) $$L_{\mathrm{InfoNCE}}=-\frac{1}{N}\sum_{i=1}^{N}\log\frac{\exp(s\left(x_{i},x_{i}^{\prime }\right)/\tau )}{\sum_{j=1}^{K}\exp\left(s\left(x_{i},x_{j}^{\prime }\right)/\tau \right)}$$

where *K* denotes the number of negative samples. Its format is very similar to NT-Xent loss (Equation (1)) but without the temporal scale factor τ. A difference between InfoNCE and NT-Xent is how to select the negative samples. In NT-Xent, the accumulated sum crosses all the negative samples in the mini-batch: there are 2N−1 negative samples. In InfoNCE, there are *K* negative samples that are pre-defined by the user or selected by a pre-defined rule (more details in [86]).

**Triplet loss**. The triplet loss is a method to measure the relative distance between three samples (i.e., triplet) [89]. Suppose we have an anchor example $x_{i}$ and an augmented sample $x_{i}^{\prime }$ (positive sample), along with a different sample $x_{j}$. Triplet loss aims to maximize the similarity between the positive pair $\left(x_{i},x_{i}^{\prime }\right)$ while minimizing the similarity between the negative pair $\left(x_{i},x_{j}\right)$. The triplet loss is formulated as

(4) $$L_{\mathrm{triplet}}=-\frac{1}{N}\sum_{i=1}^{N}\sum_{j=1,j\neq i}^{N}\max\left(0,s\left(x_{i},x_{i}^{\prime }\right)-s\left(x_{i},x_{j}\right)+\epsilon \right)$$

where s(·) is a similarity function that can be specified to task and dataset and ϵ is a hyperparameter that determines the minimum margin between positive and negative examples. To minimize the $L_{\mathrm{triplet}}$, the model is encouraged to learn a large $s(x_{i},x_{i}^{\prime })$ and a small $s(x_{i},x_{j})$. The triplet loss has been demonstrated successful in numerous tasks but it is computationally expensive. The reason is that, as shown in the equation, the nested loop requires quadratic calculation with respect to the number of training data. Thus, a smaller batch size is commonly used when using triplet loss.

### 3.8. Public Datasets

Although partial datasets are private, here, we present 51 public datasets involved in the reviewed papers that monitor physiological time series. The dataset statistics are shown in Table 4. The majority of time series data in the healthcare area are ECG, EEG, and e-ICU. Most ECG datasets contain 2 leads or 12 leads while the sampling frequency ranges from 100 Hz to 500 Hz. Compared to other data modalities, ECG signals generally have high data quality and the waveforms are easier to be recognized. Thus, contrastive learning models can achieve competitive performance in ECG-based applications such as cardiac arrhythmia detection. In terms of EEG, the datasets have a wide range of channels: from 2 electrodes to 62 electrodes. The sampling rate varies from 100 Hz to 400 Hz but the dominant frequency is 250 Hz. The most important application of EEG signals is the monitoring of sleep stages and the detection of neurological disorders (e.g., epilepsy seizure). The MASS [90] dataset utilizes 16 basic EEG channels (i.e., C3, C4, Cz, F3, F4, F7, F8, O1, O2, P3, P4, Pz, T3, T4, T5, T6) plus additional channels (Fp1, Fp2, Fpz, Fz or Oz), the specific number of the channel depending on the subset. ISRUC-SLEEP [91] dataset includes 3 sub-dataset, with 100, 8, and 10 subjects, respectively. The characteristics of ICU datasets are multi-modality and low sampling frequency. On the one hand, due to the severity of ICU patients, there are around 30 vital signs and laboratory test results. The multi-modality largely increased the complexity of ICU applications because each modality has its own pattern. On the other hand, the vital signs and lab tests are sparse and incomplete. For example, the sampling rate for Systolic BP is generally smaller than 1 Hz, and there could be days to obtain a laboratory value. The nonalignment and sparsity make machine learning models difficult to find the latent patterns of ICU activities. Thus, the current research in ICU datasets mainly focuses on relatively simple problems such as binary classification (e.g., predicting the mortality and length of stay).

**Table 4:** Summary of medical time series (e.g., physiological signal) public datasets that are used in the reviewed papers. The datasets are ordered by the data type. Further details regarding the item marked with an asterisk (*) can be found in Section 3.8.

Dataset	Data Type	Subjects	Frequency (Hz)	Channels	Task
MotionSense [92]	Acceleration, Angular velocity	24	50	12	Activity Recognition
HHAR [93]	Acceleration, Angular velocity	9	50–200	16	Activity Recognition
MobiAct [94]	Acceleration, Angular velocity	57	20	6	Activity Recognition
UCI HAR [95]	Acceleration, Angular velocity	30	50	6	Activity Recognition
PSG dataset [96]	Acceleration, HR, Steps	31	-	Acc.: 3 HR: 1 Steps: 1	Sleep study
Dutch STAN trial [97]	CTG	5681	-	-	Fetal monitoring
DiCOVA-ICASSP 2022 challenge [98]	Cough, Speech, Breath	-	44.1 k	-	COVID detection
CODE [99]	ECG	1,558,415	300–1000	12	ECG abnormalities detection
Ribeiro [100]	ECG	827		12	Automatic diagnosis of ECG
PhysioNet 2020 [101]	ECG	6877		12	ECG classification
MIT-BIH Arrhythmia [102]	ECG	47	125	2	Cardiac arrhythmia study
PhysioNet 2017 [103]	ECG	8528 (recordings)	300	1	AF(ECG) Classification
CPSC 2018 (ICBEB2018) [104]	ECG	6877	500	12	Heart diseases study
PTB [105]	ECG	290	125	14	Heart diseases study
Chapman-Shaoxing [106]	ECG	10,646	500	12	Cardiac arrhythmia study
Cardiology [107]	ECG	328		1	Arrhythmia detection and classification
PTB-XL [108]	ECG	18,869	100	12	Heart diseases study
MIT-BIH-SUP [109]	ECG	78	128	-	Supplement of supraventricular arrhythmias in MIT-BIH
Physiological Synchrony Selective Attention [110]	ECG, EEG, EDA	26	1024	EEG:32 EDA: 2 ECG:2	Attention focus study
SHHS [111,112]	ECG, EEG, EOG, EMG, SpO2, RR	9736	EEG: 125 EOG: 50 EMG: 125 ECG: 125/250 SpO2: 1 RR: 10	EEG: 2 EOG: 2 EMG: 1 ECG: 1 SpO2: 1 RR: 1	Sleep-disordered breathing study
AMIGOS [113]	ECG, EEG, GSR	40	ECG: 256 EEG: 128 GSR: 128	ECG: 4 EEG: 14 GSR: 2	Emotional states recognition
MPI LEMON [114]	EEG, fMRI	227	EEG: 2500	EEG: 62	Mind-body-emotion interactions study
PhysioNet 2016 [73]	ECG, PCG	3126	2000	2	Heart Sound Recordings classification
WESAD [115]	ECG, Acceleration, etc.	15	ECG: 700	ECG: 1	Wearable Stress and Affect Detection
SWELL [116]	ECG, Facial expressions, etc.	25	ECG: 2048	-	Work psychology study
The Fenland study [117]	ECG, HR, Acceleration, etc.	2100	-	-	Obesity, type 2 diabetes, and related metabolic disorders study
SEED [118,119]	EEG	15	200	62	Emotion Recognition
TUSZ [72]	EEG	315	250	21	Seizure study
TUAB [120]	EEG	564	250	21	ECG abnormalities study
EEG Motor Movement/ Imagery [121]	EEG	109	160	64	Motor-Imagery classification
BCI Competition IV-2A [122]	EEG	9	250	22	Motor-Imagery classification
Sleep-EDFx [123,124]	EEG	197	100	2	Sleep study
MGH Sleep [111]	EEG	2621	200	6	Sleep study
MI-2 Dataset [125]	EEG	25	200	62	Motor-Imagery classification
EPILEPSIAE [126]	EEG	275	250	-	Seizure study
UPenn Mayo Clinic’s Seizure Detection Challenge [127]	EEG (Intracranial)	4 dogs 8 human	400	16	Seizure study
DOD-H [128]	EEG (PSG data)	25	250	12	Sleep study
DOD-O [128]	EEG (PSG data)	55	250	8	Sleep study
DREAMER [129]	EEG, ECG	23	ECG: 256	ECG: 4	Affect recognition
MASS [90]	EEG, EMG, EOG	200	256	16–21 *	Sleep study
PhysioNet 2018 [130]	EEG, EOG, EMG, ECG, SaO2	1985	200	5	Diagnosis of sleep disorders
ISRUC-SLEEP [91]	EEG, EOG, EMG, ECG, SaO2	100/8/10 *	EEG: 200 EOG: 200 EMG: 200 ECG: 200 SaO2: 12.5	EEG: 6 EOG: 2 EMG: 2 ECG: 1 SaO2: 1	Sleep study
Sleep-EDF [123,124]	EEG, EOG, chin EMG	20	100	2	
MIT DriverDb [68]	ECG, EMG, EDA, PR	17	ECG: 496 EMG: 15.5 EDA: 31 PR: 31	ECG: 1 EMG: 1 EDA: 1 PR: 1	Stress detection
HiRID [79,131]	ICU	33,000+			-
eICU [80]	ICU	-	-	160 variables	-
PhysioNet 2012 [82]	ICU	12,000	-	37	Mortality prediction
MIMIC-III [78]	ICU	4000+	-	-	-
PhysioNet 2022 [124,132,133]	PCG	1568	4000	5	Heart Murmur Detection
ICBHI 2017 [134]	Respiratory sound	126	4000	1	Computational lung auscultation
LibriSpeech dataset [135]	Voice	251	16k	-	Speech Recognition
mPower data [77]	Voice, Walking kinematics	Walking: 3101	-	-	Parkinson disease study though mobile data

### 3.9. Model Transferability and Code Availability

The self-supervised contrastive learning aims to learn the representative embedding which is independent of the specific task/label. Thus, the learned models are naturally ready for transfer learning. For users who may be interested to investigate knowledge transfer, in Table 2 and Table 3, we mark the studies that have explicitly validated the transferability of their methods. Moreover, we note the implementable and reusable code can dramatically speed up the research in self-supervised contrastive learning, we also highlight the publications that publicly released their code. The accessible link to codes can be found in the original papers.

### 3.10. Evaluation Metrics

We observed that the majority of downstream tasks in the reviewed papers are classification jobs (in a broad range of medical applications). The evaluation metrics used in the papers include accuracy, precision, recall, F-1 score, Area Under Precision-Recall Curve (AUPRC), and Area Under Receiver Operating Characteristic (AUROC). In partial binary classification studies, specificity and sensitivity are also adapted to assess the self-supervised models. For a few clustering tasks, researchers employed evaluation metrics such as Normalized Mutual Information (NMI) and purity. We have summarized the model performances of the reviewed works in an extended version of Table 2 and Table 3. The extended tables also cover the GitHub code links (if applicable), data preprocessing, and technical contributions of frontier studies. Due to space limitations, we provide the most important information in this paper (Table 2 and Table 3) while storing the extended table in our GitHub repository at https://github.com/DL4mHealth/Contrastive-Learning-in-Medical-Time-Series-Survey.

## 4. Discussion and Opening Challenges

Although preliminary success has been made, self-supervised contrastive learning is still at its infant stage, especially in the context of biomedical time series. Here, we summarize the opening challenges and opportunities.

**Less guidance for augmentation design**. Data augmentation is one of the most crucial components in contrastive learning which will heavily affect the model performance. The design of sample augmentation is very complex due to the broad spectrum of temporal characteristics (sampling rate, trend, fluctuation, seasonality, etc.) across different datasets and downstream tasks. However, there is still less theoretical guidance on how to design the augmentation for time series samples. Most studies are selecting their augmentation empirically, but some augmentations may work well in one dataset/task but fail in other datasets/tasks. In addition, most of the existing sample perturbations focused on the time domain but paid less attention to the frequency domain [40] which is even more informative (evidenced by traditional signal processing [136]).

**Figure 5:**
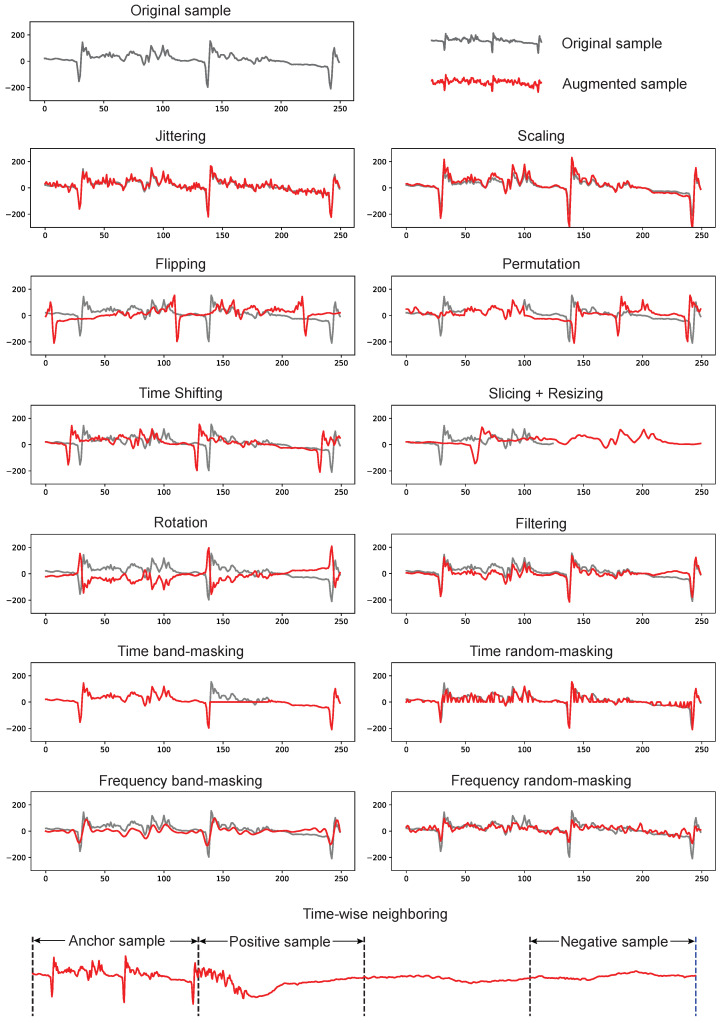
Visualization of the commonly-used augmentations for time series. In each subfigure, we present both the original sample and the augmented sample. Detailed descriptions in Section 3.4.

In this survey, we present 16 commonly used augmentations in Section 3.4 and visualize them (Figure 5) for better understanding. In future work, more innovative and effective augmentation in biomedical time series should be investigated.

**Lack of unified framework for hierarchical time series.** Different from computer vision where each image is a sample and the positive sample is certainly at the image-level, the data in medical time series is organized hierarchically. A medical time series dataset contains a number of **patients** (i.e., subjects); each patient is monitored in a number of **sessions** that are collected at a clustered time period; each session may include several **trials** where each trial is a continuous recording; every trial, generally last for seconds to minutes, can be further segmented into a series of **samples**; each sample is composed of a series of **observations** where each observation is a scalar (the readout at a single timestamp) in univariate time series.

The hierarchical organization of biomedical time series brings very high freedom in how to choose positive and negative pairs in contrastive models. However, most existing studies only applied augmentation in a single or a few levels but no framework to globally consider all the levels. Building a unified framework for contrastive representation learning for hierarchical medical time series is highly meaningful and necessary.

**Limited regression tasks.** In current self-supervised contrastive learning, most studies focus on the downstream classification tasks (such as disorder diagnosis) which require capturing the global time structure. However, few works investigate the regression task which requires more local information (i.e., the subsequence immediately prior to the to-be-predict event). The regression of medical time series plays a crucial role in health trajectory monitoring and early diagnosis of diseases. One potential reason for the scarcity of contrastive learning in regression is that there are few public datasets that provide long-term health recordings. The EHR data could be a complementary source for such studies. The effectiveness of contrastive learning needs further validation.

**Lack of scalability**. Compared to end-to-end models, contrastive learning needs to augment samples to provide measurable loss, however, the augmentation inevitably increased the number of samples which requires more computational resources [22,137]. Moreover, the larger set of negative samples can provide better contrastive performance [138]. Third, the loss functions (e.g., NT-Xent) will go through all negative samples which are more costly than traditional loss functions such as cross-entropy. Overall, for the same data size, self-supervised contrastive learning is computationally more expensive than the typical deep learning paradigms, which is harder to scale to large datasets.

**Limited ability in multimodal time series.** The mainstream of current contrastive learning models focuses on univariate time series. The augmentations are also designed based on a single-channel time series. However, in practical applications, a large proportion of medical sequences are jointly affected by multivariate signals. Thus, it is fundamentally necessary to develop contrastive learning methods that can effectively capture representative embedding from multimodal data.

**Lack of open-access diverse biomedical datasets.** The majority of existing public datasets fall in EEG, ECG, and ICU. The datasets further concentrated on a handful of tasks such as cardiovascular disease detection, sleep stage monitoring, and mortality prediction. More diverse datasets are highly demanded to improve research in medical time series.

## 5. Conclusions

This work provides a systematic review of the literature in the interdisciplinary research area of self-supervised contrastive learning and medical time series. Although this field only emerged a few years ago, dozens of studies have been published indicating the great potential of contrastive learning in addressing the limitations of sample annotation. We note that the most crucial components in contrastive learning are the design of time series augmentations, the formation of positive and negative pairs, and the choice of contrastive loss functions. In this review, we provide the most effective solutions for the above key components, which are expected to greatly benefit both computer scientists and healthcare providers in the development of contrastive learning methods. The widespread adoption of contrastive learning can largely reduce the burden of physicians by reducing the need for manual data annotation, and help enhance the efficiency and effectiveness of health systems (e.g., digital health and passive health). However, there are still some gaps in the field between the vision and current studies. We appeal to more attention from the community to address the main issues such as the guidance of augmentations and the fusion of multivariate time series. Overall, our review reveals the great potential of self-supervised contrastive learning to revolutionize the field of medical time series analysis and provide valuable insights into healthcare. We note that while this review focused on contrastive-based self-supervised representation learning, one potential future work is to summarize self-supervised generative representation learning models in medical time series.

## Figures and Tables

**Table 1:** Literature collection. We search the queries across five most popular academic databases. There are 2102 papers returned in total.

Database	Query	Articles Returned
IEEE	(((self-supervised) OR (Contrastive)) AND ((“medical time series”) OR (“physiological signal”) OR (“biomedical signal”) OR (“medical signal”) OR “biosignal”))	189
ACM		529
Scopus		60
Google Scholar		1285
MEDLINE (PubMed)	(((self-supervised) OR (“Contrastive learning”)) AND ((medical time series) OR (physiological signal) OR (biomedical signal) OR (medical signal)))	39

## Data Availability

This study does not include experimental data. However, upon acceptance, we release the implementations for time series data augmentations (12 augmentations as depicted in Figure 5; implemented by Python 3.5) at https://github.com/DL4mHealth/Contrastive-Learning-in-Medical-Time-Series-Survey.
